# Using stated preference methods to facilitate knowledge translation in implementation science

**DOI:** 10.1186/s43058-024-00554-3

**Published:** 2024-03-28

**Authors:** Whitney C. Irie, Andrew Kerkhoff, Hae-Young Kim, Elvin Geng, Ingrid Eshun-Wilson

**Affiliations:** 1 School of Social Work, Boston College, Chestnut Hill, MA USA; 2 Division of HIV, Infectious Diseases and Global Medicine Zuckerberg San Francisco General Hospital and Trauma Center, University of California, San Francisco, San Francisco, CA USA; 3 Department of Population Health at NYU Grossman School of Medicine, New York, NY USA; 4 Division of Infectious Diseases, School of Medicine, Washington University in Saint Louis, Saint Louis, MO USA; 5 Department of Global Health, Stellenbosch University, Cape Town, South Africa

**Keywords:** Knowledge translation, Stated preference research, Discrete choice experiments, Best-worst scaling

## Abstract

Enhancing the arsenal of methods available to shape implementation strategies and bolster knowledge translation is imperative. Stated preference methods, including discrete choice experiments (DCE) and best-worst scaling (BWS), rooted in economics, emerge as robust, theory-driven tools for understanding and influencing the behaviors of both recipients and providers of innovation. This commentary outlines the wide-ranging application of stated preference methods across the implementation continuum, ushering in effective knowledge translation. The prospects for utilizing these methods within implementation science encompass (1) refining and tailoring intervention and implementation strategies, (2) exploring the relative importance of implementation determinants, (3) identifying critical outcomes for key decision-makers, and 4) informing policy prioritization. Operationalizing findings from stated preference research holds the potential to precisely align health products and services with the requisites of patients, providers, communities, and policymakers, thereby realizing equitable impact.

Contributions to the literature

- Introduces stated preference methods, like discrete choice experiments (DCE) and best-worst scaling (BWS), as novel tools in implementation science for influencing behaviors of both innovation recipients and providers, offering a robust alternative to traditional methodologies.
- Demonstrates how these methods can refine intervention strategies, identify critical outcomes, and inform policy, highlighting their application across the implementation continuum.
- Showcases the potential of stated preference research to enhance equity in knowledge translation by focusing on preferences of underserved populations, thus contributing to more inclusive health services and policies.
- Provides practical examples, underscoring the methods' versatility and utility in addressing implementation science challenges.

## Background

The field of implementation science is dedicated to effectively translating evidence into real-world practice. At the heart of this pursuit lies the concept of knowledge translation, a dynamic process championed by the Canadian Institutes of Health Research, aimed at bridging the gap between research insights and tangible policy and practice outcomes [1]. The field of implementation science acknowledges the significance of incorporating input from practitioners, key decision-makers, and end-users throughout the knowledge translation cycle. However, to a certain extent, the conventional methods employed in public health have hindered the depth of integration and the resultant impact. Qualitative methods, such as interviews and focus groups, have their constraints in unveiling statistically significant associations and the generalizability of their findings. Conversely, a heavy emphasis on quantitative techniques such as surveys and electronic health record analysis may fall short of capturing the intricate individual and contextual perspectives that underpin healthcare decision-making. This has given rise to a need for an approach that surmounts the limitations of these established methods.

Stated preference research methods can address these limitations. Originating in health economics, stated preference research offers a suite of theoretical frameworks and methodological strategies tailored to understanding and influencing healthcare production and consumption. By enabling a more nuanced exploration of contextual factors and delving into the degree of association between these factors and healthcare outcomes, stated preference research methods offer a pathway beyond the limitations of conventional approaches. This article aims to highlight the potential of stated preference research as a foundational tool within the arsenal of implementation science. We describe how stated preference methods can serve multiple stages in the knowledge translation cycle. From shaping dissemination strategies that amplify the reach of vital insights to informing the blueprint and adaptation of implementation approaches, we contend that stated preference research holds the key to creating sustainable implementation initiatives that seamlessly harmonize with the intricate tapestry of context and user preferences.

## Stated preference research methods

Our commentary will center on two prominent approaches within stated preference research methods: discrete choice experiments (DCE) and best-worst scaling (BWS) exercises. These methods are grounded in random utility theory, a foundational economic principle that views individuals as logical decision-makers striving to maximize their utility. This theory assumes that when presented with a range of choices, individuals will make rational decisions by weighing trade-offs and selecting the option that yields the greatest utility, encompassing benefits, happiness, or satisfaction. Deviations from this rational behavior can often be attributed to random factors, including unobserved or unmeasured elements. Hence, these measures provide a valuable approach to evaluating the comparative acceptability, desirability, or significance of diverse attributes tied to implementation strategies and innovations—often referred to as “the seven P’s,” *encompassing pills, products, programs, practices, principles, procedures, and policies* [2].

Furthermore, stated preference research methods offer enhanced accuracy in gauging preferences and their interrelationships, surpassing standard rating scales and ranking exercises. Additionally, the diverse presentation of items or attribute levels across a series of inquiries can effectively mitigate the influence of social desirability bias, which frequently undermines outcomes from more direct questioning [3]. This approach also diminishes occurrences of straight-lining and tendencies toward favoring central options, phenomena often observed with Likert and similar rating scales.

Moreover, these stated preference techniques effectively address the issue of varied scale interpretations among respondents, a challenge particularly pronounced across cultures (called scale-use bias) [4]. They also alleviate the complications of ranking items as equals and mitigate cognitive hurdles posed by ranking lengthy lists [3, 5]. A notable advantage of stated preference methods is their ability to force participants to navigate trade-offs through a series of choice-related queries. In essence, this approach enables stated preference methods to closely mirror the way we evaluate alternatives and make selections in our everyday lives within the real world. As a result, stated preference research methods can unveil concealed perspectives that might remain concealed when employing alternative preference elicitation techniques. Notably, DCEs are frequently referred to as “conjoint analyses,” and many researchers use the terms interchangeably. However, it should be noted that traditional conjoint analyses (which originate from psychology) do not follow rational utility theory and have less well-formulated theoretical foundations and measurement methods [6]. As such, we have restricted the following discussion to the role of methods founded in rationale utility theory, namely DCEs and BWS.

## How stated preference methods contribute to the knowledge translation process

**Fig. 1:**
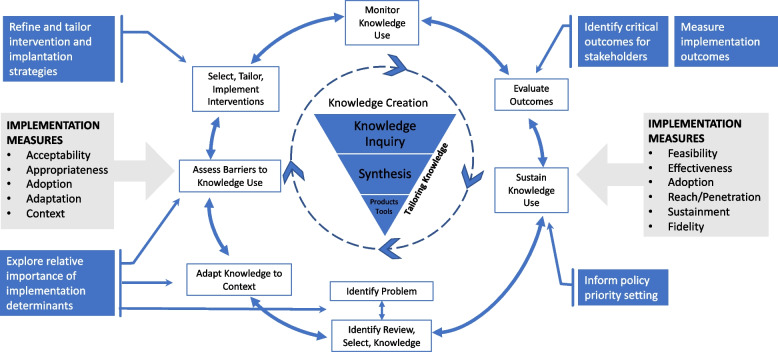
Application of stated preference research methods to knowledge translation processes in implementation science

Stated preference methods can broaden and deepen the scope of implementation outcomes by determining the relative value of features of actual or hypothetical implementation strategies and pinpointing key preference subgroups within a population. To date, stated preference research methods have most commonly been used to identify priority targets for implementation strategies by refining and tailoring intervention and implementation strategies, exploring the relative importance of implementation determinants, identifying critical outcomes for key decision-makers, and informing policy prioritization. For example, with the implementation outcome of acceptability, a study may highlight critical determinants of implementation, enact implementation strategies, and access acceptability as an implementation outcome. Using stated preference methods will help to identify and measure the mediators of acceptability. These applications within the implementation science field are summarized in Fig. 1 and are discussed in further detail below.

### Explore the relative importance of implementation determinants

Due to methodologic gaps and unclear guidance, a common challenge in implementation research and practice is understanding which implementation determinants are the most important in a given context. Applying stated preference research methods to quantify the strength of barriers and facilitators of uptake of innovations or implementation strategies permits implementers to characterize determinants more objectively (i.e., target users and key decision-makers perceptions of the features of the inner and outer setting) [5, 7–9]. For example, a BWS exercise was used to evaluate implementers’ perspectives regarding elements of the CFIR framework that were most important for the successful implementation of pharmacogenetic testing for antidepressant therapy [10]. Using BWS, authors identified features within the outer setting, inner setting, individuals, intervention, and processes most important for implementation success, demonstrating how stated preference can help identify critical determinants for future implementation efforts—by determining the relative importance (i.e., quantified value) of different implementation determinants, stated preference studies unambiguously and transparently aid in prioritizing which barriers to target and which facilitators to leverage as part of implementation strategies.

### Refine and tailor interventions and implementation strategies

Stated preference research methods are increasingly used to inform implementation strategy design, frequently combined with qualitative research methods [11–13]. For example, in Tanzania, to design a demand creation strategy to increase voluntary male medical circumcision (VMMC), a DCE informed the VMMC service delivery model regarding operating hours, provider gender, partner involvement, incentive value, and level of service integration, while qualitative research (in-depth interviews and participatory group discussions) contextualized DCE findings and additionally informed community mobilization messaging in the form of a mass media-campaign [13]. Stated preference research methods can also be embedded more formally into recognized intervention and implementation strategy development methods, including incorporation into intervention (i.e., implementation) mapping approaches or used with user-centered design participatory research [14–16]. The broad application and ability to embed DCE methods into other implementation strategy development processes demonstrate these tools’ versatility, complementariness, and utility for implementation science.

### Identify critical outcomes for key decision-makers

BWS exercises, specifically Object Case 1, are well suited for identifying which outcomes are critical for key decision-makers and how these may be aligned or misaligned. For example, researchers embedded a BWS exercise within the third round of a Delphi Process as part of a formal priority-setting exercise among patients/caretakers and healthcare providers to develop consensus-based core outcome measure domains for peritoneal dialysis research. This work identified top priorities and contrasted critical decision-makers perspectives to inform a new core set of outcome measures to improve consistency and relevance for the peritoneal dialysis research [17]. The ability of object case BWS (Case 1) to determine the relative importance of critical outcomes positions it as a key stated preference research method for defining patient-centered outcomes.

### Inform policy prioritization

A further area where stated preference methods can and have been used to inform implementation is during healthcare priority setting and policy development [16]. DCE data can directly inform policy decisions by calculating the predicted probability of uptake or choice shares, marginal rates of substitution, and willingness to pay or accept compensation [18]. This approach informed policies in Thailand for recruitment of doctors to understaffed rural areas – DCEs identified marked heterogeneity of preferences between doctors with rural compared to urban backgrounds and highlighted the importance of aligning job postings with doctors’ geographical origins as well as offering specialized training in rural areas to increase the rural healthcare workforce [19]. Such data, as well as relative preferences and relative importance ratings, can assist policymakers in decision-making, mainly when there are several implementation options to choose from and where resource constraints limit the ability to offer all options to a population.

## Future applications to facilitate knowledge translation

Several areas remain where stated preferences could further enhance knowledge translation. In a systematic review of 75 publications, the authors identified several applications of DCEs to address acceptability and appropriateness but relatively limited applications to explore questions of fidelity and feasibility [20]. While BWS could be used before implementation to determine which factors are perceived by key decision-makers to influence anticipated fidelity and feasibility most strongly, it could also be applied similarly during or after implementation as part of a comprehensive mixed-methods evaluation process to provide more significant insights as to why implementation efforts do or do not achieve their goals; however, examples to-date are lacking.

Stated preference methods also have enormous potential to aid in developing communication and dissemination strategies by identifying the most preferred communication channels, trusted messengers, and resonant messages [21]. Further, while increasingly applied as part of stated preference research in implementation science, latent-class analysis, which allows for identifying distinct preference groups that may not cluster according to sociodemographic characteristics, remains underutilized. This powerful analytic method can identify key populations with unique needs and wants, give insight into these groups’ relative size, and ultimately indicate whether multiple tailored strategies may be required to maximize reach and adoption. In addition, a method such as BWS could be used before and after implementation to identify potential implementation barriers (pre-implementation) and assess whether the strategy modified barriers as intended (post-implementation). In addition, within stated preference research, there are new techniques to advance the field, including web-based, adaptative methods that are more engaging, are tailored to each respondent, and can assess a greater number of attributes [22].

Finally, most stated preference research studies within implementation science have focused on the preferences of a single actor, namely innovation recipients or end-users (i.e., community members and patients); however, simultaneous assessment of the preferences of multiple, additional implementation actors (such as healthcare workers, community leaders, civil society groups and decision makers) can inform the design/tailoring of implementation strategies with a better overall fit to the needs and priorities of all target users and critical decision-makers through a more inclusive process. This can help achieve the “Quadruple Aim” [20] of improved experience and satisfaction for the patients/clients/innovation recipients, improved well-being of healthcare workers/innovation deliverers, improved population health, and reduced costs.

## A tool for enhancing person-centered and equity in knowledge translation

Stated preference research offers a powerful complementary approach for deciphering "what people want and need" and how their choices are shaped. This methodological avenue can substantially contribute to equity initiatives, particularly when its focus is redirected toward communities that have systematically been marginalized within conventional scientific, medical, and public health research. By dedicating preference research to the task of identifying and comprehending value-based choices and preferences, we can drive forward equity endeavors, addressing disparities that have persisted for far too long.

Much like qualitative methods, stated preference research methods center on the intricate tapestry of the human experience, unveiling the decision-making processes and underlying value systems. This enriches the core principles of qualitative techniques by broadening and deepening our grasp of personal, practical, political, and research-related insights. Embracing the elicitation of stated preferences within the realm of implementation science can act as a catalyst for expediting equity in knowledge translation.

This approach becomes a conduit for several critical aspects, including an improved prioritization of determinants driving implementation, the enhancement of implementation strategies’ alignment with users’ contextual needs, the elevation of research outcomes in order of importance, the evaluation of implementation results, and the shaping of informed policy development. By weaving stated preference research into the fabric of implementation science, we amplify the potential for achieving equity and pave the way for a more inclusive and impactful approach to disseminating knowledge equitably across diverse populations.

## Conclusion

This commentary provides a comprehensive assessment of the current applications of stated preference research in knowledge translation while also delving into potential future extensions. These methods offer a promising avenue for addressing the pressing requirement in implementation science to design initiatives that ensure dissemination and long-term viability. By harmonizing health programs, policies, or practices with their intended contexts, these methods are vital to fostering alignment and redressing health equity concerns. This is achieved through a nuanced understanding of the values and preferences of historically underserved populations, who have often received limited benefits from advancements in clinical and public health domains. Moreover, the seamless integration of stated preference research with complementary methods across all stages of implementation underscores their versatility within the field of implementation science. Their potential to enrich the breadth of implementation endeavors is evident, facilitating the delivery of health services and products meticulously tailored to the requirements of patients, healthcare providers, communities, and policymakers, ultimately culminating in outcomes of utmost equity and significance.

## Data Availability

Not applicable

## Abbreviations

BWS: Best-worst scaling
DCE: Discrete choice experiment
VMMC: Voluntary male medical circumcision
